# Pioglitazone, a PPAR-γ Activator, Stimulates BK_Ca_ but Suppresses IK_*M*_ in Hippocampal Neurons

**DOI:** 10.3389/fphar.2018.00977

**Published:** 2018-08-29

**Authors:** Tsang-Shan Chen, Ming-Chi Lai, Te-Yu Hung, Kao-Min Lin, Chin-Wei Huang, Sheng-Nan Wu

**Affiliations:** ^1^Department of Neurology, Tainan Sin-Lau Hospital, Tainan, Taiwan; ^2^Department of Pediatrics, Chi-Mei Medical Center, Tainan, Taiwan; ^3^Department of Pediatric Neurology, Chiayi Christian Hospital, Chiayi, Taiwan; ^4^Department of Neurology, College of Medicine, National Cheng Kung University, Tainan, Taiwan; ^5^Department of Physiology, College of Medicine, National Cheng Kung University, Tainan, Taiwan

**Keywords:** pioglitazone, Ca^2+^-activated K^+^ current, large-conductance Ca^2+^-activated K^+^ channel, M-type K^+^ current, hippocampal neuron

## Abstract

Pioglitazone (PIO), a thiazolidinedone, was reported to stimulate peroxisome proliferator-activated receptor-γ (PPAR-γ) with anti-inflammatory, anti-proliferative, anti-diabetic, and antidepressive activities. However, whether this compound exerts any perturbations on Ca^2+^-activated K^+^ and M-type K^+^ currents in central neurons remains largely unresolved. In this study, we investigated the effects of PIO on these potassium currents in hippocampal neurons (mHippoE-14). In whole-cell current recordings, the presence of PIO (10 μM) increased the amplitude of Ca^2+^-activated K^+^ current [*I*_K(Ca)_] in mHippoE-14 cells. PIO-induced stimulation of *I*_K(Ca)_ observed in these cells was reversed by subsequent addition of paxilline, yet not by TRAM-39 or apamin. In inside-out current recordings, PIO applied to the bath concentration-dependently increased the activity of large-conductance Ca^2+^-activated K^+^ (BK_Ca_) channels with an EC_50_ value of 7.6 μM. Its activation of BK_Ca_ channels in mHippoE-14 cells was voltage-dependent and accompanied by both a lengthening in mean open time and a shortening in slow component of mean closed time. The activation curve of BK_Ca_ channels after addition of PIO was shifted to less depolarized potential without any change in the gating charge. PIO also suppressed the amplitude of M-type K^+^ currents inherently in mHippoE-14 neurons. Taken together, in addition to its agonistic action on PPAR-γ, PIO-induced perturbation of these potassium channels may be responsible for its widely pharmacological actions on hippocampal neurons.

## Introduction

The family of PPARs is a type II nucleus receptor. They have been recognized to play essential roles in the regulation of cellular differentiation, development, and metabolism. There are three distinct isotypes in PPARs including α, γ, and β/δ (Kersten et al., 2000). Among them, PPAR-γγ plays an important role in fatty acid storage and glucose metabolism, expressing in endothelium, vascular muscle, and cells of the innate immune system (Tontonoz and Spiegelman, 2008). Two isoforms of PPAR-γ are detected in the human and in the mouse, i.e., PPAR-γ1 and PPAR-γ2. The two PPAR-γ isoforms show a distinct expression pattern; that is, PPAR-γ1 is abundantly expressed, while PPAR-γ2 was reported to be restricted to white and brown adipose tissue under physiological conditions.

PPAR-γ heterodimerizes with retinoid X receptor (RXR) and binds to specific PPAR response element. PPAR-γ is activated by natural and synthetic ligand. Natural ligands include fatty acids and prostanoids (e.g., 15-deoxy-δ12,14-prostaglandin J2). TZDs are synthetic PPAR-γ ligands, including rosiglitazone and pioglitazone. They are also type II diabetes drugs approved by U.S. Food and Drug Administration. There is growing evidence that the agonist of PPAR-γ might have neuroprotective potential in CNS diseases such as stroke, Parkinson’s disease and depression (Kapadia et al., 2008; Schlachetzki and Winkler, 2015; Li et al., 2018).

There were the experimental results showing the electrophysiological effects of troglitazone and PIO in African green monkey kidney cells. They found that ATP-sensitive K^+^ (K_ATP_) channels, which closed when intracellular [ATP]/[ADP] ratio elevated and membrane depolarization, were inhibited by troglitazone but not by PIO (Sunaga et al., 1999). In a previous report, troglitazone has been demonstrated to suppress K_ATP_ activity in ventromedial hypothalamus neurons (Asano et al., 1999). However, it was noted that FDA-approved TZDs only included rosiglitazone and PIO but not troglitazone. Until now, investigations on TZDs’ electrophysiological effect in the brain is inconclusive.

The mHippoE-14 hippocampal cell line is known to possess the characteristics of embryonic hippocampal neurons and enables accurate *in vitro* assays for use in the discovery, development, and validation of new therapeutics targeted to central nervous system diseases and disorders, including obesity, stress, reproduction, and metabolic disorders (Gingerich et al., 2010; Lazniewska et al., 2013; Evans et al., 2016). Long-term treatment with PIO has been previously reported to influence hippocampal function (Blalock et al., 2010). However, to our knowledge, no studies concerning the biophysical and pharmacological properties of membrane ionic currents in these mHippoE-14 hippocampal cells have been thoroughly studied, although several previous reports have shown some effects of PIO on K^+^ and Ca^2+^ currents in muscle cells and Ca^2+^ currents in cultured hippocampal neurons (Zhang et al., 1994; Pancani et al., 2009; Gu et al., 2014).

The large-conductance Ca^2+^-activated K^+^ (BK_Ca_) channels (maxi-K channels, K_Ca_1.1, *KCNMA1*, *Slo1*) have the largest single-channel conductance of all K^+^ selective channels and can be synergistically activated by membrane depolarization and elevation of intracellular Ca^2+^. They have been described to be functionally expressed in hippocampal neurons (Li et al., 2002; Huang et al., 2007; Wu et al., 2017). Another interesting paper has previously shown the function of Ca^2+^-activated K^+^ channels in hippocampal pyramidal neurons (Storm, 1987). Specifically, a previous report has demonstrated the ability of PPAR-γ inhibitor to either increase or decrease the activity of BK_Ca_ channels (Wu et al., 2000). On the other hand, the KCNQ2, KCNQ3, and KCNQ5 genes are known to encode the core subunits of K_V_7.2, K_V_7.3, and K_V_7.5 channels, respectively. The increased activity of these K^+^ channels has been demonstrated to generate the M-type K^+^ current [*I*_K(M)_] which is a slowly activating and deactivating current suppressed by stimulation of muscarinic receptors (Brown and Yu, 2000; Hsu et al., 2014). Most of *I*_K(M)_ in neurons are made by heterologously expressed K_V_7.2/7.3 channels. Targeting *I*_K(M)_ is growingly recognized to be valuable as an adjunctive regimen for the treatment of many neurological disorders. However, whether PIO exerts any actions on these types of ionic currents remains largely unexplored.

In this study, we attempted to explore the effect of PIO on potassium currents, mainly Ca^2+^-activated K^+^ current [*I*_K(Ca)_], and M-type K^+^ current [*I*_K(M)_] observed in mHippoE-14 hippocampal neurons. Our findings revealed that PIO is able to stimulate the activity of BK_Ca_ channels and to suppress *I*_K(M)_ with different potency. Apart from its agonism on PPAR-γ activation, these actions on potassium currents may significantly have an impact on electrical behaviors of hippocampal neurons.

## Materials and Methods

Pioglitazone (PIO, C_19_H_2_ON_2_O_3_S, 5-[[4-[2-(5-ethylpyridin-2-yl)ethoxy]phenyl]methyl]-1,3-thiazolidine-2,4-dione) was obtained from Takeda Pharmaceutical, Co., Ltd. (Tokyo, Japan), 4-aminopyridine, flupirtine maleate, linopirdine, paxilline, tetraethylammonium chloride and tobutamide were from Sigma-Aldrich (St. Louis, MO, United States), TRAM-39 was from Tocris (Bristol, United Kingdom) and apamin was from Alomone Labs (Jerusalem, Israel). Chlorotoxin was kindly provided by Dr. Woei-Jer Chuang (Department of Biochemistry, National Cheng Kung University Medical College, Tainan, Taiwan). Pioglitazone was dissolved in dimethyl sulfoxide (less than 0.1%) and made immediately prior to experiments. Unless stated otherwise, tissue culture media, fetal bovine serum, L-glutamine, penicillin–streptomycin, fungizone, and trypsin/EDTA were obtained from Invitrogen, Corp. (Carlsbad, CA, United States). All other chemicals including CsCl and CsOH were of the best available quality, mostly at analytical grades. The twice-distilled water was de-ionized through a Milli-Q water purification system (APS Water Services, Inc., Van Nuys, CA, United States).

The composition of bath solution (i.e., normal Tyrode’s solution) was as follows (in mM): NaCl 136.5, KCl 5.4, CaCl_2_ 1.8, MgCl_2_ 0.53, glucose 5.5, and HEPES-NaOH buffer 5 (pH 7.4). To record *I*_*K*(Ca)_ or *I*_K(M)_, the recording pipettes were backfilled with a solution consisting of K-aspartate 130, KCl 20, KH_2_PO_4_ 1, MgCl_2_ 1, Na_2_ATP 3, Na_2_GTP 0.1, EGTA 0.1, and HEPES-KOH buffer 5 (pH 7.2). For the recordings of BK_Ca_-channel activity or whole-cell *I*_K(M)_, high K^+^-bathing solution was composed of the following (in mM): KCl 145, MgCl_2_ 0.53, and HEPES-KOH 5 (pH 7.4), and the pipette was filled with a solution (in mM): KCl 145, MgCl_2_ 2, and HEPES-KOH 5 (pH 7.2). The value of p/4Ca^2+^ concentration was calculated assuming a dissociation constant for EGTA and Ca^2+^ (at pH 7.2) of 0.1 μM^1^. For example, to provide 0.1 μM Ca^2+^ in the bath solution, 0.5 mM CaCl_2_ and 1 mM EGTA were added.

### Cell Preparation

Embryonic mouse hippocampal cell line (mHippoE-14; CLU198) was obtained from Cedarlane CELLutions Biosystems, Inc. (Burlington, ON, Canada) (Gingerich et al., 2010). Cells were grown as a monolayer culture in 50-ml plastic culture flasks in a humidifier environment of 5% CO_2_/95% air at 37°C. They were maintained at a density of 10^6^/ml in 5 ml of Dulbecco’s modified Eagle’s medium supplemented with 10% heat-inactivated fetal bovine serum (v/v) and 2 mM L-glutamine. The medium was refreshed every 2 days to maintain a healthy cell population. Under our experimental conditions, the presence of neuritis and varicosities during cell preparations was often observed. The patch clamp experiments were performed 5 or 6 days after cells were subcultured (60–80% confluence).

### Electrophysiological Measurements

Mouse hippocampal neurons (mHippoE-14) were harvested with 1% trypsin/EDTA solution prior to each experiment and a portion of detached cells was thereafter transferred to a recording chamber mounted on the stage of a CKX-41 inverted fluorescent microscope (Olympus, Tokyo, Japan) coupled to a digital video system (DCR-TRV30; Sony, Japan) with a magnification of up to 1500×. They were immersed at room temperature (20–25°C) in normal Tyrode’s solution containing 1.8 mM CaCl_2_. Patch pipettes were made from Kimax-51 glass capillaries (#34500; Kimble, Vineland, NJ, United States) using a PP-830 (Narishige, Tokyo, Japan) or P-97 micropipette puller (Sutter, Novato, CA, United States), and their tips were then fire-polished with an MF-83 microforge (Narishige). The recording pipettes used had a resistance of 3–5 MΩ as they were immersed in different solutions described above. Patch-clamp recordings were made in whole-cell, cell-attached, or inside-out configuration by means of an RK-400 (Bio-Logic, Claix, France) or Axopatch 200B patch amplifier (Molecular Devices, Sunnyvale, CA, United States) (Huang et al., 2018; Lai et al., 2018). Liquid junctional potential was commonly nulled immediately before establishment of the seal.

### Data Recordings

The signals consisting of voltage and current tracings were stored online in an ASUSPRO-BU401LG computer (ASUS, Taipei, Taiwan) at 10 kHz connected through a Digidata 1440 digitizer (Molecular Devices) which was driven by pCLAMP 10.2 software (Molecular Devices). Current signals were low-pass filtered at 3 kHz. The data achieved during each experiment were analyzed off-line using different kinds of analytical tools including LabChart 7.0 program (AD Instruments; Gerin, Tainan, Taiwan), OriginPro 2016 (OriginLab, Northampton, MA, United States) and custom-made macro procedures built under Microsoft Excel 2013 (Redmond, WA, United States). Through digital-to-analog conversion, the gapped voltage-step protocols with either rectangular or ramp pulses created from pCLAMP 10.2 were employed to evaluate the steady-state activation or inactivation curve for different types of ion currents.

### Single-Channel Analyses

Single amplitudes of BK_Ca_-channel currents were analyzed with pCLAMP 10.2 (Molecular Devices). Multi-gaussian adjustments of the amplitude distributions among channels or mean-variance histograms were implemented to determine single-channel events accurately. The number of functional active channels seen in each patch was taken as the maximum number of channels simultaneously open under conditions of maximum open probability. The channel open probabilities were evaluated using an iterative process to minimize χ^2^ values, as calculated with an adequately large number of independent observations. Single-channel conductance of the channel taken in the absence or presence of PIO was determined by linear regression using mean values of current amplitudes collected at different levels of holding potentials. The lifetime distributions of open or closed states were satisfactorily fitted with single or two exponentials, and the distributions are illustrated in logarithmically scaled bin width. The mean-variance analysis obtained with or without addition of PIO was also made for determination of single-channel opening events (Wu et al., 2003).

To calculate percentage increase of PIO on the probability of BK_Ca_-channel openings, inside-out current recordings were made, bath medium contained 0.1 μM Ca^2+^, and the potential was set at +60 mV. BK_Ca_-channel activity in the presence of 100 μM PIO was taken to be 100% and those in different concentration (0.1–30 μM) of PIO were then compared. The PIO concentration needed to increase 50% of channel open probability was determined using the following multi-parameter logistic equation:

$$y=\frac{E_{\max}\times {\left[C\right]}^{\mathrm{nH}}}{{\mathrm{EC}}_{50}^{\mathrm{nH}}\times {\left[C\right]}^{\mathrm{nH}}},$$

where [C] is the PIO concentration, EC_50_ and *n_H_* are the concentration required for a 50% increase of channel activity and the Hill coefficient, respectively; and *E*_max_ is the PIO-induced maximal increase in the probability of BK_Ca_ channels that would be open.

To determine effects of PIO on the activation curve of BK_Ca_ channels, the ramp pulse from 0 to +80 mV with a duration of 1 s were repetitively delivered from pCLAMP 10.2 program through digital-to-analog conversion. The activation curves evoked in response to ramp pulses were calculated by averaging current traces in responses to 20 voltage ramps and dividing each point of mean current by single-channel amplitude at each potential when the leakage component was corrected. The linear passive leak currents were subtracted using a P/4 regimen. The activation curve of BK_Ca_ channels (i.e., the relative open probability versus membrane potential) taken with or without addition of PIO (10 μM) was fitted by the Boltzmann function:

$$\mathrm{Relative open probability}=\frac{P_{O(\max)}}{1+\exp[-(V-V_{1/2)\mathrm{qF}/\mathrm{RT}}]},$$

where *P*_*O*(max)_ is the maximal open probability of BK_Ca_-channel openings taken during the exposure to 10 μM PIO at the level of +80 mV, *V*_1/2_ the voltage at which half-maximal activation occurs, *q* the apparent gating charge, *F* Faraday’s constant, R the universal gas constant and *T* the absolute temperature.

### Statistical Analyses

The values are expressed as the means ± SEM with sample sizes (*n*) indicating the number of cells from which the data were obtained, and error bars are plotted as SEM. The linear or non-linear curve-fitting to the data sets was performed with the aid of either Excel 2013 (i.e., Excel’s Solver add-in) or OriginPro 2016. The paired or unpaired Student’s *t*-test, or one-way analysis of variance (ANOVA) followed by *post hoc* Fisher’s least-significance difference test for multiple-group comparisons, were used for the statistical evaluation of differences among means. Non-parametric Kruskal–Wallis test was used, as the assumption of normality underlying ANOVA was violated. Statistical analyses were made using SPSS version 22.0 (IBM, Corp., Armonk, NY, United States). Statistical significance was determined at a *P*-value of <0.05.

## Results

### Effect of PIO on Whole-Cell Ca^2+^-Activated K^+^ Current (I_K(Ca)_) in mHippoE-14 Hippocampal Neurons

**FIGURE 1:**
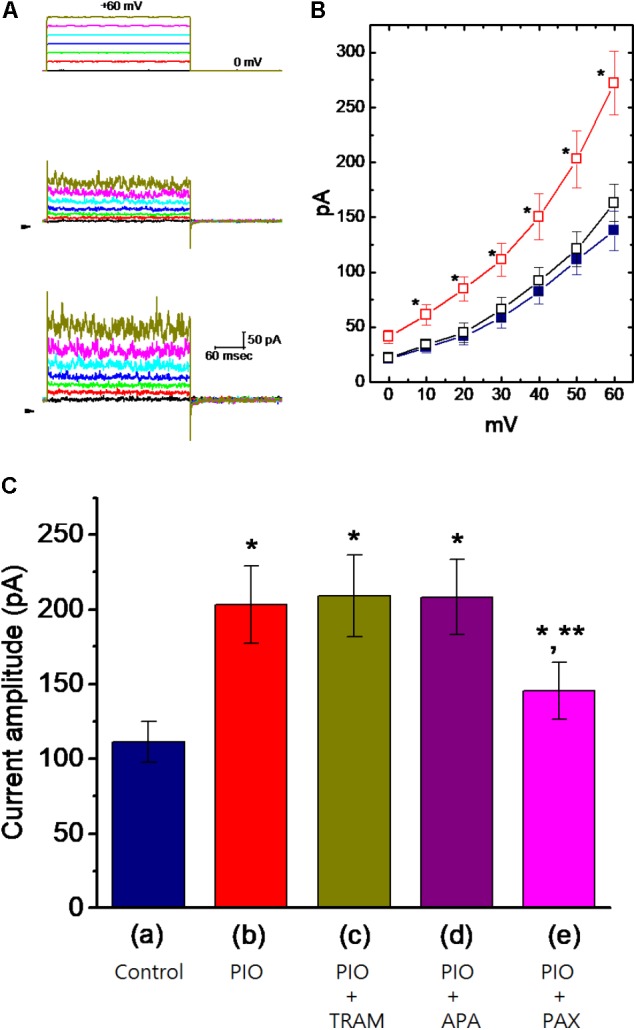
Effect of PIO on whole-cell Ca^2+^-activated K^+^ current (*I*_K(Ca)_) in mHippoE-14 hippocampal neurons. In these experiments, cells were bathed in normal Tyrode’s solution, the composition of which was described under Section “Materials and Methods.” The recording pipette was filled with K^+^-containing solution. **(A)** Superimposed *I*_K(Ca)_ traces obtained in the control (middle part) and during cell exposure to 10 μM PIO (bottom part). The upper part indicates the voltage protocol applied, and arrowheads are zero current level. **(B)** Averaged *I–V* relationships of *I*_K(Ca)_ obtained in the control (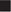), during the exposure (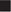) to 10 μM PIO and after washout (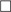) of PIO (mean ± SEM; *n* = 11 for each point). ^∗^Significantly different from control groups taken at the same level of voltage pulse. **(C)** Bar graph showing summary of the effect of PIO, PIO plus TRAM-39, PIO plus apamin, and PIO plus paxilline, and PIO plus tolbutamide on *I*_K(Ca)_ amplitude (mean ± SEM; *n* = 10–12 for each bar). Current amplitude was measured at +50 mV. (a) Control; (b) 10 μM PIO; (c) 10 μM PIO plus 3 μM TRAM-39; (d) 10 μM PIO plus 200 nM apamin; (e) 10 μM PIO plus 1 μM paxilline; (f) 10 μM PIO plus 30 μM tolbutamide. ^∗^Significantly different from control (*P* < 0.05) and ^∗∗^significantly different from PIO alone group (*P* < 0.05) (*n* = 9–10 for each bar).

Whole-cell current recordings were initially made to evaluate whether PIO can exert any effects on ionic currents in these cells. In these experiments, cells were immersed in normal Tyrode’s solution containing 1.8 mM CaCl_2_, and the recording pipette was filled with K^+^-containing solution. To inactivate most of other K^+^ currents, the examined cells were held at 0 mV and the depolarizing voltages ranging between 0 and +60 mV were applied. Of interest, as cells were exposed to PIO at a concentration of 10 μM, current amplitudes were readily elevated throughout the entire depolarizing voltages examined (**Figures 1A,B**). For example, as the examined cell was depolarized from 0 to +50 mV, current amplitude was progressively increased to 203 ± 26 pA from a control value of 111 ± 14 pA (*n* = 11, *P* < 0.05). After washout of the compound, current amplitude was returned to 121 ± 16 pA (*n* = 11). Likewise, whole-cell ionic conductance measured at the voltages ranging between +40 and +60 mV was significantly increased from 2.8 ± 0.2 to 6.1.2 ± 0.4 nS (*n* = 11, *P* < 0.05).

The nature of PIO-induced simulation of whole-cell *I*_K(Ca)_ in mHippoE-14 hippocampal neurons was further evaluated. As illustrated in **Figure 1C**, in continued presence of 10 μM PIO, further addition of TRAM-39 (3 μM) or apamin (200 nM) was not found to have any measurable effects on PIO-mediated increase of *I*_*K*(Ca)_ in these cells. TRAM-39 and apamin are inhibitors of intermediate- (IK_Ca_) and small-conductance Ca^2+^-activated K^+^ (SK_Ca_) channels, respectively. Similarly, chlorotoxin (1 μM), an inhibitor of Cl^−^ channels, failed to counteract PIO-stimulated increase of *I*_K(Ca)_. However, in continued presence of PIO, subsequent application of paxilline (1 μM) known to suppress the activity of BK_Ca_ channels was effective at counteracting the inhibition of *I*_K(Ca)_ caused by PIO. The PIO-mediated increase of *I*_K(Ca)_ was little affected by further addition of 30 μM tolbutamide, an inhibitor of K_ATP_ channels. The data thus prompted us to suggest that PIO-induced stimulation of whole-cell *I*_K(Ca)_ could predominantly arise from its effects on the activity of BK_Ca_ channels, not on those of IK_Ca_ or SK_Ca_ channels.

### PIO Enhances BK_Ca_-Channel Activity Measured From mHippoE-14 Hippocampal Neurons

**FIGURE 2:**
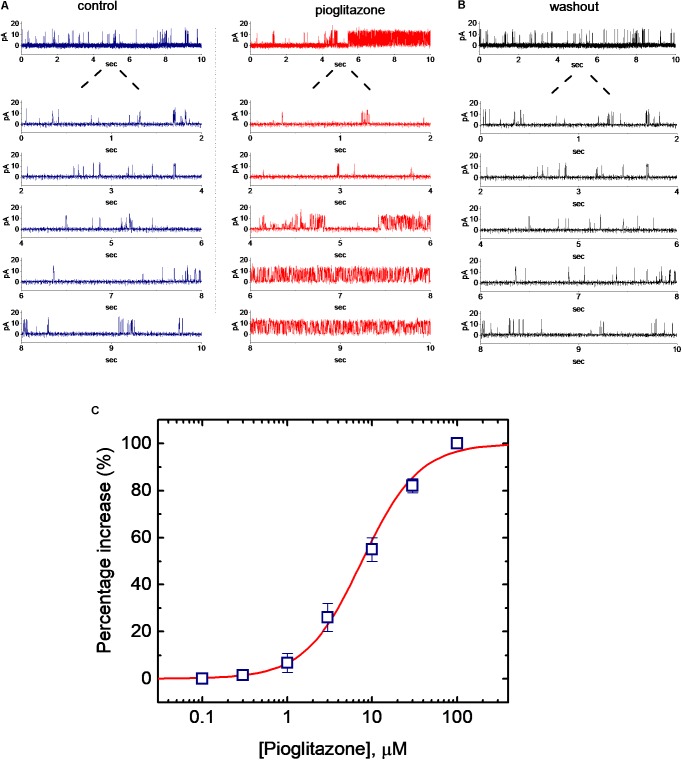
Effect of PIO on BK_Ca_ channel activity in mHippoE-14 hippocampal neurons. **(A)** Original current traces of BK_Ca_ channels obtained in the absence (left) and presence (right) of 10 μM pioglitazone (PIO). The examined cells were bathed in symmetrical K^+^ solution (145 mM). Under inside-out current recordings, the potential was held at +60 mV and bath medium contained 0.1 μM Ca^2+^. The upward deflection represents the opening event of the channel. The lower part indicates the expanded trace recorded from the uppermost part in the control and during exposure to PIO. **(B)** BK_Ca_-channel trace obtained after washout of PIO. **(C)** Concentration-dependent increase in channel open probability (mean ± SEM; *n* = 9–11 for each point). Channel activity measured at +60 mV during the exposure to 100 μM PIO was taken to be 100%. The values for EC_50_, Hill coefficient and maximal percentage increase of BK_Ca_ channels in the presence of PIO were 7.6 μM 1.3 and 100%, respectively.

In order to further assess whether and how PIO can alter the activity and kinetics of BK_Ca_ channels in these cells, single-channel current recordings were further performed. In these experiments, cells were bathed in symmetrical K^+^ concentration (145 mM) and bath medium contained 0.1 μM Ca^2+^. Under inside-out configuration, the examined cell was held at the level of +60 mV. As PIO at a concentration of 10 μM was applied to the bath, channel activity was drastically raised (**Figure 2A**), as evidenced from the results showing that addition of PIO (10 μM) increased the probability of channel openings significantly from 0.072 ± 0.004 to 0.132 ± 0.007 (*n* = 11, *P* < 0.05). After washout of the compound, channel activity returned to 0.081 ± 0.004 (*n* = 8) (**Figure 2B**).

Concentration-dependent stimulation of BK_Ca_-channel activity in mHippoE-14 hippocampal neurons was further evaluated. The relationship between the PIO concentration and the percentage increase of BK_Ca_ channels was derived and is hence illustrated in **Figure 2C**. To measure channel activity taken with or without addition of different PIO concentrations (0.1–100 μM), each detached patch was held at +60 mV. As the intracellular surface of excised patch was exposed to PIO, the probability of BK_Ca_-channel openings was progressively raised in a concentration-dependent manner. Based on a modified form of Hill equation, the half-maximal concentration (EC_50_) required for stimulatory effect of PIO on channel open probability was calculated to be 7.6 ± 0.3 μM, and it at a concentration of 100 μM increased almost all of channel activity in these cells.

**FIGURE 3:**
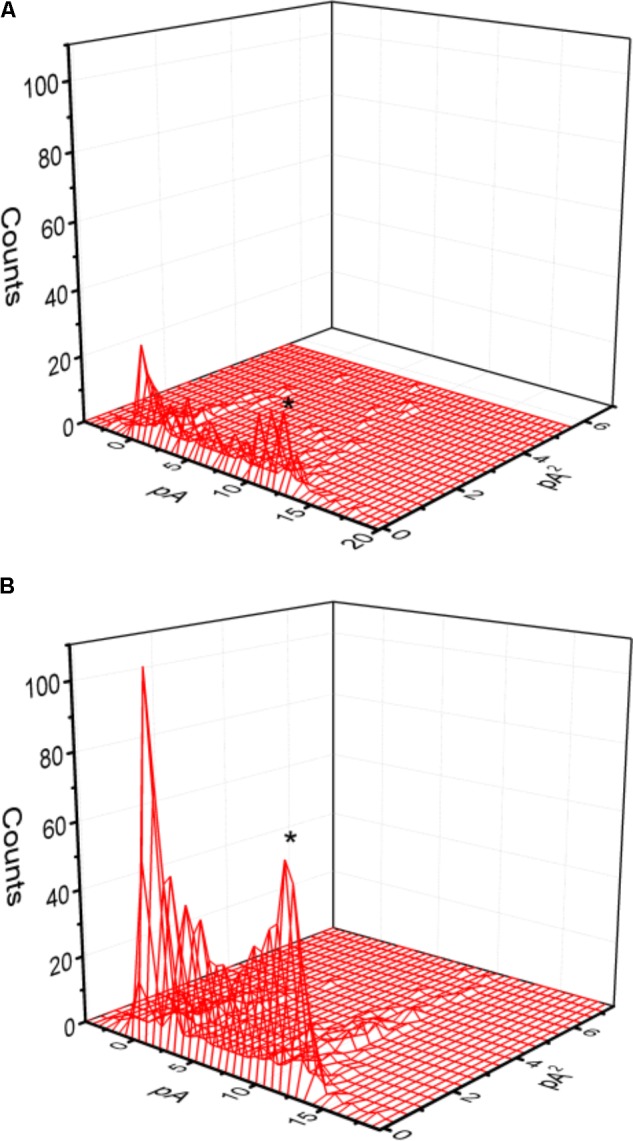
Mean-variance histogram of BK_Ca_ channels taken from the absence **(A)** and presence **(B)** of 10 μM PIO. The examined cells were bathed in symmetrical K^+^ solution, bath medium contained 0.1 μM Ca^2+^ and the holding potential was set at +60 mV. The closed state corresponds to the peak at 0 pA. The mean currents (indicated by asterisk) in **(A,B)** were 10.9 and 10.7 pA, respectively.

Mean-variance analysis for single BK_Ca_-channel currents was further performed to evaluate whether PIO has any effect on single-channel amplitude once the open level was detected. As shown in **Figure 3**, the single-channel amplitude was not noted to differ significantly between the absence and presence of PIO (10 μM), despite its ability to elevate channel open probability.

### Effect of PIO on the Kinetic Behavior of BK_Ca_ Channels

**FIGURE 4:**
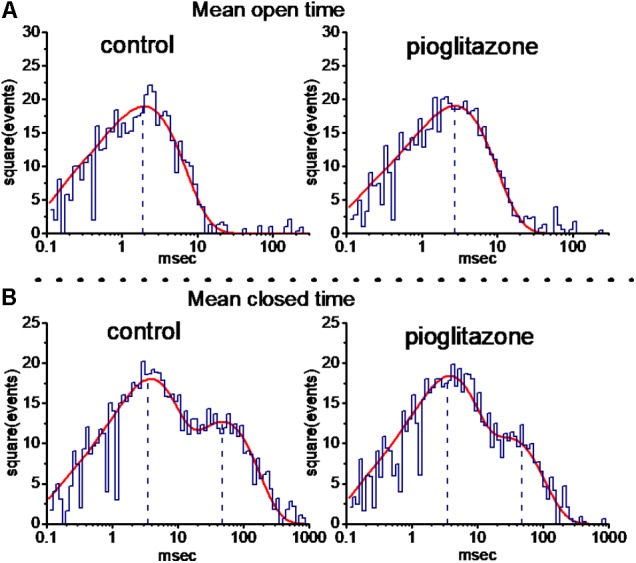
Effect of PIO on mean open- **(A)** and closed-time **(B)** histograms of BK_Ca_ channels recorded from mHippoE-14 hippocampal neurons. The holding potential was set at +60 mV, and inside-out configuration was performed. In control (left side), the open-time histogram of the channel was fitted by a single exponential function (indicated by red smooth line) with a mean open time of 1.9 ms, while the closed-time histogram was by a sum of a two-exponential function with a mean closed time of 3.5 and 47.5 ms. After addition of 10 μM PIO (right side), the mean open time was increased to 2.7 ms, and the slow component of closed time was shortened to 28.7 ms; however, minimal change in the fast component of closed time (i.e., 3.4 ms) in the presence of this compound. Of note, the abscissa and ordinate in each histogram indicate the logarithm of open or closed time (ms) and the square root of even number, respectively. Data were taken from a measurement of 100 channel openings. The vertical black dashed lines are placed at the values of mean open or closed time for BK_Ca_ channels.

Pioglitazone-induced increase of BK_Ca_-channel activity may result from different types of single-channel kinetic behavior. As such, its effects on mean open and closed times of these channels recorded from mHippoE-14 hippocampal neurons were further examined and analyzed. As depicted in **Figure 4**, in an excised patch, the open-time histogram of BK_Ca_ channels at the level of +60 mV was fitted by a single exponential with mean open time of 1.9 ± 0.2 ms (*n* = 9). Addition of 10 μM PIO prolonged the lifetime of the open state to 2.7 ± 0.3 ms (*n* = 9, *P* < 0.05). On the other hand, the closed-time histogram of BK_Ca_ channels measured at the same level was necessarily fitted by a two-exponential curve with mean closed time of 3.5 ± 0.2 and 47.5 ± 1.3 ms (*n* = 9). Addition of 10 μM PIO decreased the slow component of mean closed time to 28.7 ± 1.1 ms (*n* = 9, *P* < 0.05), while it had minimal change in the fast component of mean closed time (3.4 ± 0.2 ms, *n* = 9, *P* > 0.05). Therefore, it becomes clear that stimulatory effect of PIO on BK_Ca_-channel activity in these cells is predominantly attributable to a lengthening in channel open time and a decrease in the slow component of channel closed time, although no change in single-channel amplitude was clearly seen.

### Lack of Pioglitazone Effect on Single-Channel Conductance of BK_Ca_ Channels

**FIGURE 5:**
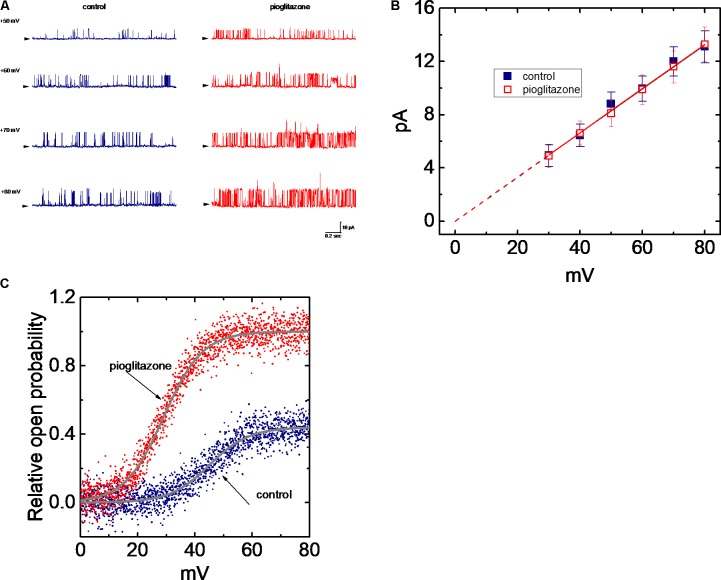
Effect of PIO on the *I–V* relation of BK_Ca_ channels in mHippoE-14 hippocampal neurons. The experiments on BK_Ca_ channels were conducted with symmetrical K^+^-rich concentration (145 mM). Under inside-out configuration, the potential was held at +60 mV and bath medium medium contained 0.1 μM Ca^2+^. **(A)** Original current traces obtained in the control and during exposure to 10 μM PIO. The labels in the rightmost side indicate the holding potential applied. Arrowhead in each trace corresponds to zero current level, and the upper deflection indicates the opening event of the channel. In **(B)**, the single-channel conductance in the absence (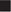) and presence (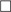) of 10 μM PIO is nearly identical. Each point represents mean ± SEM (*n* = 9–10). The dashed red lines obtained with or without addition of PIO are pointed toward the values of the reversal potential (i.e., 0.0 ± 0.1 mV, *n* = 8). **(C)** The relationship between relative open probability of BK_Ca_ channels and membrane potential obtained with or without addition of 10 μM PIO. The ramp pulses were applied from 0 to +80 mV with a duration of 1 s. Under inside-out current recordings, PIO (10 μM) was applied to the intracellular surface of the excised patch. The smooth lines represent the best fit to the Boltzmann equation as detailed in Section “Materials and Methods.”

We also measured the amplitude of single BK_Ca_ channels at a series of voltages ranging between +30 and +80 mV. Throughout the voltage range examined, the *I–V* relationships of BK_Ca_ channels taken with or without addition of PIO (10 μM) were analyzed and then compared (**Figures 5A,B**). In the control, fitting these single-channel amplitudes with a linear regression revealed BK_Ca_ channels of 165 ± 6 pS (*n* = 12). The value for these channels obtained in the absence of PIO did not differ significantly from that (166 ± 7 pS, *n* = 13, *P* > 0.05) during exposure to this agent. It is clear from the results that this compound applied intracellularly was unable to modify single-channel conductance, although it significantly raised the probability of BK_Ca_ channels that would be open.

### Effect of PIO on the Activation Curve of BK_Ca_ Channels

**Figure 5C** shows the activation curve of BK_Ca_ channels taken with or without addition of PIO (10 μM). In this set of experiments, the curves were achieved by use of digitized voltage-ramp protocols together with digital-to-analog conversion. The long-lasting ramp pulses were repetitively delivered from 0 to +80 mV with a duration of 1 s. The plots of relative open probability of BK_Ca_ channels as a function of membrane potential was derived and then fitted with the Boltzmann function as described in Section “Materials and Methods.” In control (i.e., in the absence of PIO), *P*_O(max)_ = 0.44 ± 0.03, *V*_1/2_ = 45.1 ± 0.8 mV and *q* = 3.6 ± 0.3 *e* (*n* = 13), whereas in the presence of PIO, *P*_*O*(max)_ = 0.99 ± 0.01, *V*_1/2_ = 28.9 ± 0.8 mV and *q* = 3.7 ± 0.2 *e* (*n* = 11). Therefore, the experimental results showed that PIO not only produced a 2.3-fold increase in the maximal open probability of the channel, but it also significantly shifted the activation curve to a lower membrane potential by approximately 16 mV; however, minimal change in the *q* value (i.e., gating charge) of the curve was seen in its presence. Therefore, the presence of PIO is capable of enhancing the activity of BK_Ca_ channels in a voltage-dependent fashion, despite no effect on single-channel conductance.

### Effect of PIO on M-Type K^+^ Current (I_*K*(*M*)_) in mHippoE-14 Hippocampal Neurons

**FIGURE 6:**
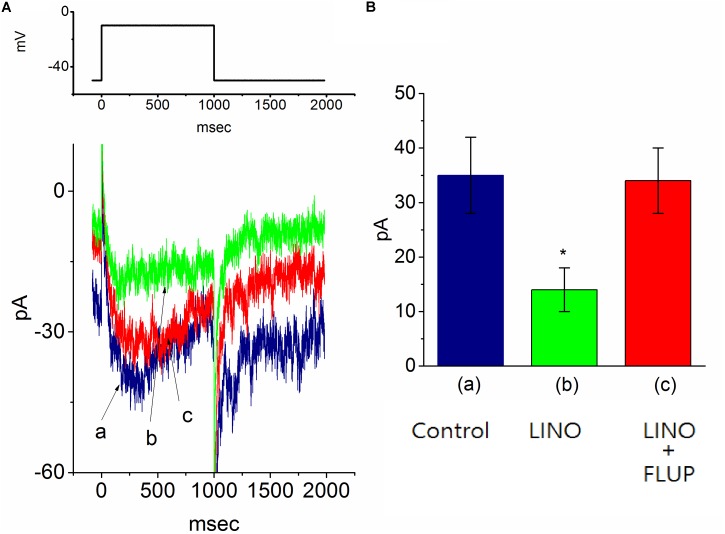
Effect of linopirdine and linopirdine plus flupirtine on the amplitude of M-type K^+^ current [*I*_*K*(*M*)_] in mHippoE-14 hippocampal neurons. In this set of experiments, cells were bathed in high K^+^, Ca^2+^-free solution and the recording pipette was filled with K^+^-containing solution. **(A)** Superimposed *I*_K(M)_ traces obtained in the control (a) and during the exposure to 10 μM linopirdine (b), and 10 μM linopirdine plus 10 μM flupirtine (c). The upper part indicates the voltage protocol used. **(B)** Bar graph showing the effect of linopirdine and linopirdine plus flupirtine on *I*_*K*(*M*)_ amplitude (mean ± SEM; *n* = 9 for each bar). ^∗^Significantly different from control (*P* < 0.05). LINO, linopirdine; FLUP, flupirtine.

**FIGURE 7:**
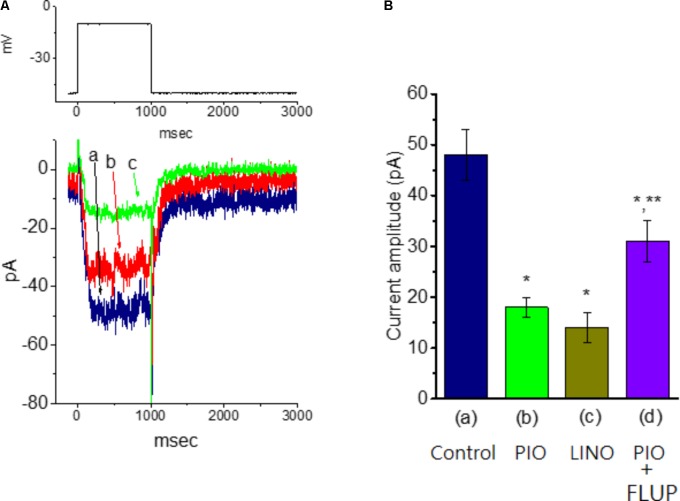
Effect of PIO on *I*_*K*(*M*)_ amplitude in mHippoE-14 hippocampal neurons. These experiments were conducted in cells bathed in high K^+^, Ca^2+^-free solution and the recording pipette was filled with K^+^-containing solution. **(A)** Superimposed *I*_*K*(*M*)_ traces obtained in the absence (a) and presence of 3 μM (b), and 10 μM PIO (c). The upper part indicates the voltage protocol used. **(B)** Bar graph showing the effect of PIO, linopirdine, and PIO plus flupirtine on *I*_*K*(*M*)_ amplitude (mean ± SEM; *n* = 9–11 for each bar). The *I*_*K*(*M*)_ amplitude elicited by membrane depolarization from –50 to –10 mV was measured. (a) Control; (b) 10 μM PIO; (c) 10 μM linopirdine; (d) 10 μM PIO plus 10 μM flupirtine. ^∗^Significantly different from control (*P* < 0.05) and ^∗∗^significantly different from PIO (10 μM) alone group (*P* < 0.05). LINO, linopirdine; FLUP, flupirtine.

In another set of experiments, we explored the possible effect of PIO on *I*_*K*(*M*)_ observed in these cells. The examined cells were bathed in high-K^+^, Ca^2+^-free solution and the recording pipette was filled with K^+^-containing solution. As shown in **Figure 6**, when the cell was depolarized from −50 to −10 mV with a duration of 1 s, K^+^ inward current with the slowly activating and deactivating properties was readily evoked. This K^+^ current elicited by long-lasting membrane depolarization was sensitive to inhibition by linopirdine (10 μM), yet not by either 4-aminopyridine (1 mM) or tetraethylammonium (10 mM), and it was hence identified as an *I*_*K*(*M*)_ (Sankaranarayanan and Simasko, 1996; Selyanko et al., 1999; Huang et al., 2008). Linopirdine is recognized as a selective blocker of *I*_K(M)_ (Huang et al., 2008). Notably, as cells were exposed to PIO, the *I*_*K*(*M*)_ amplitude evoked in response to depolarizing pulse from −50 to −10 mV was progressively diminished, as evidenced by the data showing that addition of 10 μM PIO caused a significant reduction in current amplitude from 48.1 ± 5.1 to 17.8 ± 1.9 pA (*n* = 11, *P* < 0.05). As *I*_*K*(*M*)_ elicited in response to membrane depolarization was noted to be suppressed by addition of 10 μM linopirdine. In continued presence linopirdine, further application of 10 μM flupirtine significantly reversed linopirdine-induced inhibition of *I*_*K*(*M*)_ in mHippoE-14 neurons (**Figure 6**). Moreover, in continued presence of 10 μM PIO, subsequent addition of 10 μM flupirtine, an activator of *I*_*K*(*M*)_ (Wu et al., 2012), was found to reverse its inhibition of *I*_*K*(*M*)_ (**Figure 7**). Therefore, it is possible that the presence of PIO suppressed the amplitude of *I*_*K*(*M*)_ effectively in mHippoE-14 hippocampal neurons.

## Discussion

The pharmacological effects of PIO on ionic currents (i.e., *I*_K(Ca)_ and *I*_*K*(*M*)_) seen in mHippoE-14 hippocampal neurons were described in this study. Application of PIO did not simply raise the amplitude of whole-cell *I*_K(Ca)_. It produced a significant increase in the probability of BK_Ca_ channels that would be open with no change in single-channel amplitude. Our findings reflect that PIO is capable of enhancing BK_*Ca*_-channel activity in a concentration-, voltage-, and state-dependent manner. Moreover, a leftward shift in the midpoint of the activation curve for the probability of channel openings was detected during the exposure to PIO.

In our study, PIO-mediated increase in BK_Ca_-channel activity in mHippoE-14 hippocampal neurons is not due to an increase in single-channel amplitude because of the absence of notable difference in single-channel conductance taken with or without addition of PIO. However, a notable increase in mean open time accompanied by the shortening in slow component of mean closed time may predominantly contribute to PIO-mediated activation of BK_Ca_ channels. Moreover, the present results showed that the presence of PIO was capable of shifting the activation curve of BK_Ca_ to less depolarized potential, although no change in the gating charge for this activation was seen. This compound interacted with BK_Ca_ channels in a voltage-dependent fashion and its action would depend on the pre-existing level of membrane potential, the concentration of PIO used, or both.

Previous studies have demonstrates the ability of different TZDs to modulate the activity of K_ATP_ channels (Sunaga et al., 1999). The intracellular solution used in our whole-cell recordings contained 3 mM ATP, a value thought to suppress the activity of functional K_ATP_ channels adequately. The PIO-mediated increase of *I*_K(Ca)_ was little affected by further addition of 30 μM tolbutamide, an inhibitor of K_ATP_ channels. Neither TRAM-39 nor apamin countered PIO-mediated increase of *I*_K(Ca)_, while further addition of paxilline was effective at reversing it. Therefore, PIO-induced increase of K^+^ outward currents described in this study is unlikely to be predominantly linked to the stimulation of K_ATP_, IK_Ca_ or SK_Ca_ channels, although these channels might be functionally active in hippocampal neurons.

Finding from the present results tends to be consistent with previous observations showing that different TZDs can regulate the activity of BK_Ca_ channels (Wu et al., 2000; Li et al., 2002). Several studies have previously demonstrated that troglitazone and PIO might differentially perturb ionic currents in isolated cardiac myocytes and in pancreatic β cells (Ikeda and Watanabe, 1998; Sunaga et al., 1999). Therefore, the ability of PIO to stimulate BK_Ca_ channels as well as to suppress *I*_*K*(*M*)_ amplitude observed in the present study appears to be necessarily noted with caution in relation to its use as an agonist of PPAR-γ activation.

In continued presence of PIO, neither TRAM-39 nor apamin produced any effects on its stimulation of *I*_K(Ca)_. TRAM-39 and apamin are known to block the intermediate- and small-conductance Ca^2+^-activated K^+^ channels, respectively. The observed effect of PIO on whole-cell *I*_K(Ca)_ inherently in mHippoE-14 hippocampal neurons clearly did not involve the stimulation of small- and intermediate-conductance Ca^2+^-activated K^+^ channels. The activation of BK_Ca_ channels by PIO could be predominantly responsible for the increase of macroscopic *I*_K(Ca)_ amplitude.

As far as the resting membrane potential is concerned, membrane hyperpolarization due to PIO-induced increase of BK_Ca_ channels could be potentially offset by its suppression *I*_*K*(*M*)_. On the other hand, increased activity of BK_Ca_ channels would be expected to hasten the repolarization of neuronal action potential and to facilitate *I*_Na_ recovery. As a result, apart from stimulation of the nuclear receptor PPAR-γ, to what extent fluctuations in the resting membrane potential accompanied by shortening of neuronal action potential caused by PIO contributes to its pharmacological actions (e.g., insulin-sensitizing effects) remain to be further delineated.

One previous study reported that a peak plasma level of 3.4 μM for total PIO is present following a maximal PIO dose of 45 mg (Budde et al., 2003) and PIO was considered as “harmless at therapeutic concentrations” and it’s not likely that normally dosed PIO can strongly alter cardiac electrogenesis in healthy individuals (Kistamás et al., 2013). Although, the EC50 value required for PIO to stimulate BKCa-channel activity is relatively higher in our study. However, the present results showed that the presence of PIO was capable of shifting the activation curve of BKCa to less depolarized potential. This compound was capable of interacting with BKCa channels in a voltage-dependent fashion and its action would depend on the pre-existing level of membrane potential, the concentration of PIO used, or both. The effects reported herein are likely to be the relevant at therapeutic doses. Such discrepancy remains to be further delineated in the near future.

In summary, both stimulation of BK_Ca_ channels and inhibition of *I*_*K*(*M*)_ caused by PIO may synergistically act to influence the electrical behavior of hippocampal neurons.

## Author Contributions

T-SC, M-CL, C-WH, and S-NW conducted the experiments and data interpretation. T-SC, M-CL, T-YH, and C-WH analyzed the data and assisted in data interpretation. K-ML, C-WH, and S-NW interpreted study data and assisted in statistical analyses. T-SC, M-CL, C-WH, and S-NW conceptualized and designed the study, interpreted study data, and revised the manuscript. C-WH and S-NW contributed to qualify as the senior authors.

## Conflict of Interest Statement

The authors declare that the research was conducted in the absence of any commercial or financial relationships that could be construed as a potential conflict of interest.

## Footnotes

1 http://maxchelator.stanford.edu/CaEGTA-TS.htm

## Abbreviations

BK_Ca_ channel: large-conductance Ca^2+^-activated K^+^ channel
EC_50_: the concentration required for half-maximal stimulation
FDA: Food and Drug Administration
*I–V*: current versus voltage
IK_Ca_ channel: intermediate-conductance Ca^2+^-activated K^+^ channel
*I*_K(Ca)_: Ca^2+^-activated K^+^ current
K_ATP_: channel
ATP-sensitive: K^+^ channel
PIO: pioglitazone
PPAR-γ: peroxisome proliferator-activated receptor-γ
SEM: standard error of mean
SK_Ca_ channel: small-conductance Ca^2+^-activated K^+^ channel
TZD: thiazolidinedione.
